# Cavity Quantum Electrodynamics Complete Active Space Configuration Interaction Theory

**DOI:** 10.1021/acs.jctc.3c01207

**Published:** 2024-01-30

**Authors:** Nam Vu, Daniel Mejia-Rodriguez, Nicholas P. Bauman, Ajay Panyala, Erdal Mutlu, Niranjan Govind, Jonathan J. Foley

**Affiliations:** † Department of Chemistry, University of North Carolina Charlotte, 9201 University City Blvd., Charlotte, North Carolina 28223, United States; ‡ Physical and Computational Sciences Directorate, Pacific Northwest National Laboratory, Richland, Washington 99352, United States; ¶ Department of Chemistry, University of Washington, Seattle, Washington 98195, United States

## Abstract

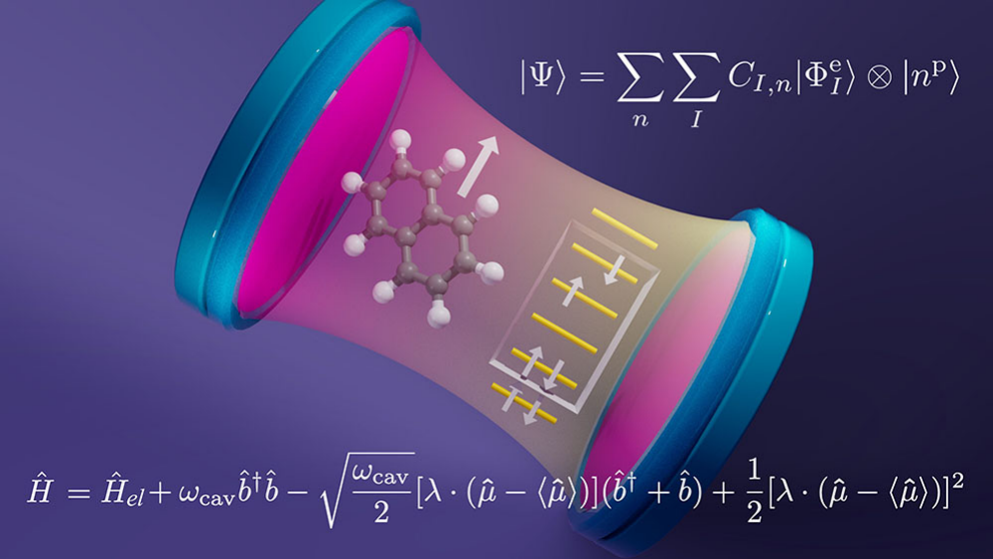

Polariton chemistry has attracted great attention as a potential route to modify chemical structure, properties, and reactivity through strong interactions among molecular electronic, vibrational, or rovibrational degrees of freedom. A rigorous theoretical treatment of molecular polaritons requires the treatment of matter and photon degrees of freedom on equal quantum mechanical footing. In the limit of molecular electronic strong or ultrastrong coupling to one or a few molecules, it is desirable to treat the molecular electronic degrees of freedom using the tools of *ab initio* quantum chemistry, yielding an approach we refer to as *ab initio* cavity quantum electrodynamics, where the photon degrees of freedom are treated at the level of cavity quantum electrodynamics. Here, we present an approach called Cavity Quantum Electrodynamics Complete Active Space Configuration Interaction theory to provide ground- and excited-state polaritonic surfaces with a balanced description of strong correlation effects among electronic and photonic degrees of freedom. This method provides a platform for *ab initio* cavity quantum electrodynamics when both strong electron correlation and strong light–matter coupling are important and is an important step toward computational approaches that yield multiple polaritonic potential energy surfaces and couplings that can be leveraged for *ab initio* molecular dynamics simulations of polariton chemistry.

## Introduction

The field of polariton chemistry has excited the imagination of chemists, physicists, and materials scientists who have demonstrated many compelling ways that strong coupling between light and molecules can affect chemical properties and dynamics.^1−29^ The theoretical challenges in polaritonic chemistry bridge most domains of chemical physics, including *ab initio* quantum chemistry, cavity quantum electrodynamics, computational electrodynamics, statistical thermodynamics, and rate theories as pointed out by a recent review and perspective articles on theoretical advances in polaritonic chemistry.^30−33^ In this work, we introduce a method called Cavity Quantum Electrodynamics Complete Active Space Configuration Interaction theory (QED-CASCI) that provides a variational route to ground- and excited-state polariton states and assesses its accuracy against several model systems for which we can provide numerically exact benchmarks. QED-CASCI provides an important theoretical toolkit at the intersection of *ab initio* quantum chemistry and cavity quantum electrodynamics, which we will generally refer to here as *ab initio* cavity quantum electrodynamics, that should be able to inherit many of the favorable properties that have enabled CASCI to become a workhorse method in simulating *ab initio* molecular dynamics on multiple potential energy surfaces, namely, smooth potential energy surfaces,^34^ size consistent and size intensive vertical excitation energies,^35^ the ability to formulate analytic nuclear derivatives and derivative couplings,^36^ and the ability to formulate relatively simple corrections to incorporate dynamic correlation.^37^

The goal of *ab initio* cavity quantum electrodynamics methodologies in general is to provide a detailed and rigorous description of the molecular energy eigenstates coupled to quantized photon states that are treated within the framework of cavity quantum electrodynamics. Ideally, these approaches should provide reliable accuracy across coupling regimes, i.e., spanning weak, strong, and ultrastrong coupling. There are at least two complementary approaches to this problem: so-called parametrized CQED methods and self-consistent CQED methods. The former parametrized approach involves solving two Schrödinger equations in series: a first for the molecular system alone using traditional tools of *ab initio* quantum chemistry, and the second for the coupled molecular-photonic system that is parametrized by the solutions to the molecular problem.^30,38,39^ On the other hand, the self-consistent approach involves augmenting *ab initio* quantum chemistry methods to directly include coupling to photonic degrees of freedom. Such approaches have included quantum electrodynamics generalizations of density functional theory (QEDFT^32,40−45^ and QED-DFT^46−48^), real-time^40,41,49−52^ and linear-response^46,53,54^ formulations of QED-TDDFT, configuration interaction (QED-CIS),^55^ cavity QED extension of second-order Møller-Plesset perturbation theory and the algebraic diagrammatic construction,^56,57^ coupled cluster (QED-CC),^48,58−60^ variational QED-2-RDM methods,^61^ and diffusion Monte Carlo.^62^ To the best of our knowledge, this is the first formulation of QED-CASCI.

A central part of CASCI (and other active-space or multireference configuration interaction calculations) includes performing an FCI expansion within an active space defined by a number of active electrons *N*_*el*_ with all possible excitations within a number of active orbitals *N*_*orbs*_, (*N*_*el*_, *N*_*orbs*_), where just as in FCI, the number of determinants scales factorially with the number of active electrons and orbitals.^63^ A number of algorithmic advances have pushed the limits of the sizes of these calculations. A major advance in direct CI algorithms in the 1990s due to Olsen and co-workers led to active spaces on the order of (10,10) with roughly 1 billion determinants.^64^ The current state of the art in massively parallel direct active space CI algorithms can treat active space sizes of (22,22) with roughly 1 trillion determinants.^65^ There have also been several exciting developments that can push even beyond this (22,22) active space size through various means of reducing the number of determinants that are included in the CAS wave function, including the Selected CI approach using a basis of tensor product states,^66^ adaptive sampling CI,^67^ the so-called iCISCF approach,^68^ and active learning approaches to CI.^69^ Additionally, a family of active-space approaches where the 2-particle reduced density matrix (rather than the many-electron wave function) is variationally optimized can also enable active spaces beyond this (22,22) limit,^70−73^ including CASSCF-like calculations with active spaces as large as (64,64).^74^ In this work, we adopt a serial implementation of the direct CI approach of Olsen and co-workers to *ab initio* QED using the Pauli–Fierz Hamiltonian within the dipole and Born–Oppenheimer approximation. We present two formulations of QED-CASCI: PN-QED-CASCI, in which the photonic space is represented on the basis of photon number (PN) states, and CS-QED-CASCI, in which the photonic space is represented on the coherent state (CS) basis. Our serial implementation of this method can routinely handle (12,12) active spaces for the electronic subsystem with at least 100 photonic states.

## Theory

We will discuss QED-CASCI as an approach to the energy eigenstates of the Pauli–Fierz (PF) Hamiltonian^32,33,75−77^ within the Born–Oppenheimer and dipole approximations. The Pauli–Fierz Hamiltonian for a molecular system coupled to a single photonic mode in atomic units as:
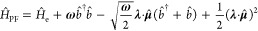
1Here, *Ĥ*_e_ is the standard molecular electronic Hamiltonian within the Born–Oppenheimer approximation, **ω** is the cavity photon frequency, **μ̂** is the molecular dipole operator, **λ** is a coupling vector, and *b̂*^†^, *b̂* are creation and annihilation operators for the cavity photonic mode, respectively. The coupling vector can be written in terms of the cavity volume, . The light–matter coupling in the Pauli–Fierz Hamiltonian may also be written in terms of the vector potential operator **A**_0_(*b̂*^†^ + *b̂*); for readers familiar with this notation, we note that , we can write .^30,33^ Here, we have limited our considerations to a single cavity mode, but the above Hamiltonian is easily generalized for multiple modes (which leads to increased dimensionality) as discussed in prior work.^55,58^ The second term *Ĥ*_cav_ = ***ω**b̂*^†^*b̂* represents the Hamiltonian for the bare cavity mode, which is a harmonic oscillator with fundamental frequency **ω**. The last two terms are the bilinear coupling,  and dipole self-energy terms , respectively. We will assume a Cartesian coordinate system in which **λ** and **μ̂** will have *x*, *y*, and *z* components. The molecular dipole operator **μ̂** has both electronic and nuclear contributions, i.e., **μ̂** = **μ̂**_e_ + **μ**_n_. In the Born–Oppenheimer approximation, the nuclear contribution is a constant for a given geometry. In the remainder of the paper, we will adopt the notation *d̂* = **λ**·**μ̂** for simplicity, and in cases where we require only the electronic part of this operator, we will denote it as *d̂*_e_ and we will denote the nuclear contribution as *d*_n_.

The formulation of mean-field theories of eq 1 (e.g., QED-Hartree–Fock, QED-HF) is aided by transformation to the coherent-state basis,^33,78^
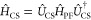
2This coherent-state transformation is defined as:
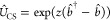
3where *z* is a parameter defined such that ⟨Φ_0_^e^|*Û*_CS_(*Ĥ*_cav_ + *Ĥ*_blc_ + *Ĥ*_dse_)*Û*_CS_^†^|Φ_0_^e^⟩ is a diagonal operator, where |Φ_0_^e^⟩ denotes the electronic reference determinant. In particular, this holds when
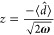
4The term ⟨*d̂*⟩ = **λ**·⟨**μ̂**⟩ in eq 4 is computed from the expectation value of the molecular dipole moment which typically comes from a modified Hartree–Fock calculation that includes cavity effects, e.g., QED-HF.^55,78^ Applying this transformation with this choice of *z* to eq 1 yields the Pauli–Fierz Hamiltonian in the coherent state basis:
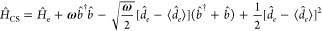
5We will formulate QED-CASCI for both eq 1 and eq 5, which we will denote as PN-QED-CASCI and CS-QED-CASCI to make reference to the photon number (PN) basis and coherent state (CS) basis for the photonic degrees of freedom, respectively. Although we will consider the reference states to be |*R*⟩ = |Φ_0_^e^⟩⊗|0^p^⟩ in both cases, we note that there are two key differences between these formulations. The first difference between these formulations is that the reference for CS-QED-CASCI formally includes an infinite number of photon occupation states through
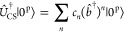
6where the right-hand side of eq 6 defines a coherent state wave function for a photon.^30,33,77,78^ The second difference is that the electronic reference determinant is written in terms of the canonical molecular orbitals for the PN-QED-CASCI formulation and in terms of the QED-HF orbitals in the CS-QED-CASCI formulations. This latter difference represents a particular choice of a single-electron orbital basis; there are many other valid choices of this orbital basis^34^ that can be explored in future work. One implication of this choice, however, is that the orbital basis of CS-QED-CASCI depends on the details of the cavity, while the orbital basis of PN-QED-CASCI does not. The independence of the orbital basis of the PN-QED-CASCI formalism may impart additional flexibility to model systems with arbitrary cavity shapes and mode densities; for example, plasmonic cavities could be modeled with this formalism provided that the Hamiltonian was adapted to include longitudinal Coulombic interactions with the charges involved in plasmon excitations. The CS-QED-CASCI formalism can also be formulated with a cavity-independent orbital basis, and future work will provide a more systematic study of the behavior of different choices of orbital basis for these methods.

**Figure 1:**
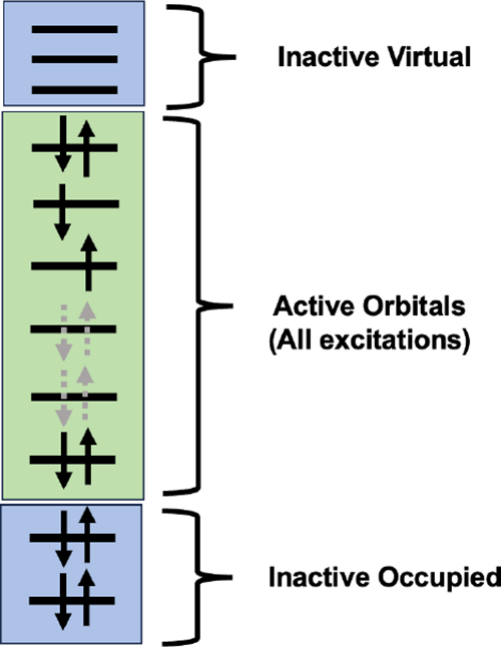
Schematic of the electronic contribution to the QED-CASCI wave function.

A general correlated wave function for a many-electron system coupled to a single-mode cavity can take the form:
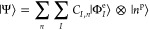
7where |Φ_*I*_^e^⟩ represents a determinant of electronic orbitals, |*n*^p^⟩ is a photon-number state corresponding to *n* photons in the cavity mode, and *C*_*I*,*n*_ is an expansion coefficient. In the CASCI ansatz for the electronic subspace, a subset of active electrons and orbitals are identified, where a full CI expansion is performed within that active space. We use the convention (*N*_el_, *N*_orb_) to denote an active space consisting *N*_orb_ active orbitals including the *m* highest-energy occupied orbitals containing *N*_el_ active electrons and the (*N*_orb_ – *m*) remaining lowest-energy unoccupied orbitals. A schematic of one possible determinant in a (6,6) active space is shown in Figure 1. As previously discussed, because we perform an FCI expansion within the active space, the number of electronic determinants *N*_det_ in eq 7 scales factorially with the number of active electrons and orbitals. However, *N*_det_ is insensitive to the total size of the single-electron orbital basis used for a given active space size. We can then see that the key to having a tractable CASCI (and QED-CASCI) method relies on having a reasonably large active space. We also have additional scaling of the number of electronic–photonic product states in eq 7 by maximum photon occupation. For example, if we restrict the maximum photon occupation state to be 1 (so that the photon basis states include |0^p^⟩ and |1^p^⟩, then we will have twice as many configurations in eq 7 as compared to a cavity-free calculation with the same active space, and the resulting Hamiltonian matrix will be four times as large. In general, the size of the QED-CASCI wave function grows as (*N*^p^ + 1)*N*_det_ where *N*^p^ denotes the maximum photon occupation state. We will use the nomenclature PN-QED-CASCI(*N*_el_, *N*_orb_)-*N*^p^/CS-QED-CASCI(*N*_el_, *N*_orb_)-*N*^p^ to denote the electronic active space and photonic space truncation in the photon number and coherent state formulations of QED-CASCI, respectively.

The variational QED-CASCI problem in the photon number or coherent state representation formally involves building and diagonalizing the matrix representation of eq 1 or eq 5, respectively, on the basis of states shown in eq 7. The CI matrix in both representations with a maximum photon occupation of *N*^p^ will read:
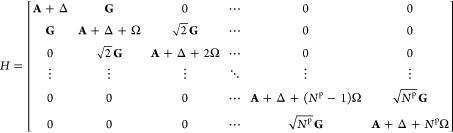
8Here the matrix elements of **A** are:
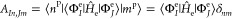
9which are common integrals in CI calculations. For PN-QED-CASCI, these and all subsequent integrals are performed in the canonical MO basis, whereas for CS-QED-CASCI, we transform these and all subsequent integrals to the (orthonormal) QED-HF basis. The elements of **Δ** and **G** involve slightly different integrals for the photon number formulation as compared to those of the coherent state formulation. The matrix elements of **Δ** in the photon number formulation are:
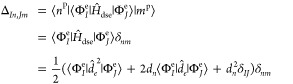
10and for the coherent state formulation are:
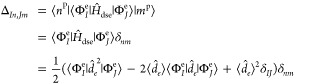
11The elements of **G** are:
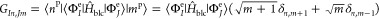
12where in the photon number formulation,
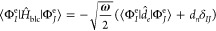
13and in the coherent state formulation,
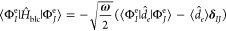
14Finally, the elements of **Ω** for both formulations are given by:
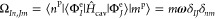
15The block structure shown in eq 8 reflects the Kronecker deltas in the expressions for the matrix elements. Each of these integrals over many-electron determinants can be simplified using the Slater–Condon rules^79^ and expressed in terms of 1- and 2-electron integrals in the canonical MO basis for the PN-QED-CASCI formulation or in the QED-HF MO basis for the CS-QED-CASCI formulation; we make use of these expressions in the subsequent section on the Implementation Details.

## Implementation Details

For most CI problems, it is not practical to build and store the Hamiltonian matrix. Rather, one typically employs so-called direct CI schemes that employ an iterative eigensolver for one or a few states where the requisite Hamiltonian matrix elements are computed on the fly.^63,64^ Here, we outline the adaptation of such a direct approach to the QED-CASCI problem specifically for the Hamiltonian in the coherent state basis (eq 5); as noted in eqs 10–14, one can follow the details with a few key substitutions in the integrals to obtain the implementation for the photon number basis (eq 1).

The Hamiltonian in eq 5 can be rewritten as:
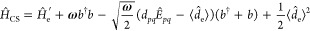
16where *Ê*_*pq*_ is a generator of the unitary group:
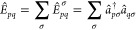
17The generator for a spin σ (σ = α, β) is expressed in terms of the Fermionic creation operator *â*_*pσ*_^†^ and the Fermionic annihilation operator *â*_*qσ*_. The modified electronic Hamiltonian is given by:
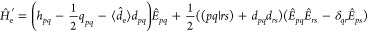
18The terms *h*_*pq*_ and (*pq*|*rs*) denote the standard 1- and 2-electron integrals over spatial orbitals in Chemist’s notation, and *d*_*pq*_ and *q*_*pq*_ represent modified electric dipole and electric quadrupole integrals, which are given by:
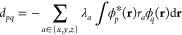
19
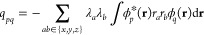
20Here, λ_*a*_ is a Cartesian component of **λ**, and *r*_*a*_ is a Cartesian component of the electronic position operator [e.g., for **r** = (*x*, *y*, *z*), *r*_*x*_ = *x*]. Next, we define the modified 1- and 2-electron integrals as follows:
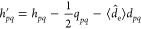
21
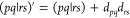
22

The eigenvalues of the Hamiltonian 16 in a CI space are obtained from an iterative process using Davidson algorithm,^80^ where the most time-consuming step at each iteration is to compute the **σ** vector:
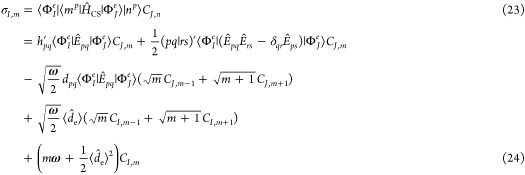
23
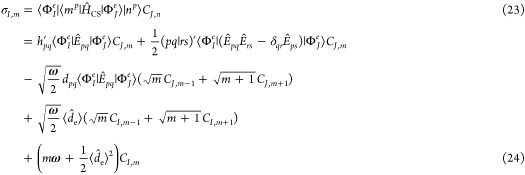


Following Handy^81^ we express a Slater determinant as a combination of an alpha string and a beta string:
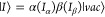
25where an alpha/beta string is an ordered product of creation operators for alpha/beta molecular spin orbitals. Applying the Olsen method,^82^**σ** is rewritten as:
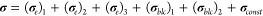
26where
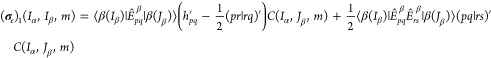
27
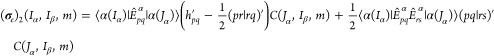
28
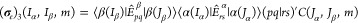
29
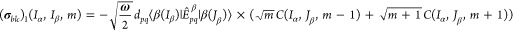
30
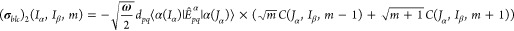
31
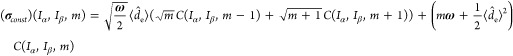
32We have adapted the algorithm to evaluate the first two terms from ref (82). The sigma vectors corresponding to bilinear coupling terms can be calculated in a fashion similar to those of the one-electron parts of (**σ**_*e*_)_1_ and (**σ**_*e*_)_2_. The last term is simply a product of scalars and the CI vector. The construction of **σ**_3_ is the most time-consuming step in the Olsen method. We use the algorithm for building **σ**_3_ described in ref (65) by rewriting eq 29 as follows:
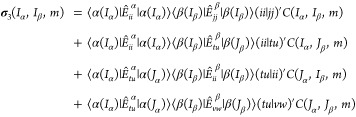
33where the indices *i*, *j* represent inactive orbitals and indices *t*, *u*, *v*, *w* represent active orbitals. For the first three terms of the σ_3_ expression, we have either *I*_α_ = *J*_α_ or *I*_β_ = *J*_β_ (or both). These identities and the fact that the summation on the right-hand side of eq 33 includes all inactive orbitals allow us to simplify the loop structures and easily adapt a vectorized algorithm. The first term contains the same string indices present in the left-hand side of the equation; thus, its contribution to the sigma vector is simply a product of the trial vector and a scalar. The second term and the third term can be vectorized similarly to σ_1_ and σ_2_ (note that the identities *I*_α_ = *J*_α_ or *I*_β_ = *J*_β_ also exist in σ_1_ and σ_2_). The algorithm to build the second term is shown in Algorithm S1 in the Supporting Information. It is less trivial to adapt the vectorized algorithm for the last term because no such identities exist for this term.^63^ To vectorize the last term, we insert a resolution of identity into the first bracket of this term and get:
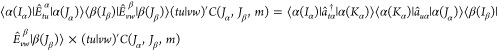
34The string |α(*K*_α_)⟩ has one electron less than the reference state. Then the construction of σ_3_ (for the active part) becomes a sequence of three operations:
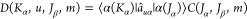
35
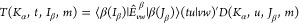
36
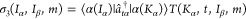
37Among these steps, the inner loop of eq 36 can be evaluated as matrix–matrix multiplication.

The Davidson algorithm requires exact or approximate diagonal elements of the Hamiltonian matrix. In our work, we adapt the exact formulation of determinant energy in ref (84) with additional terms belonging to the PF Hamiltonian. The expression for the energy of each determinant reads:
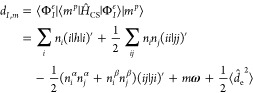
38where *n*_*i*_^α^ = 0 or 1 is the occupation number of alpha spin in spatial orbital ϕ_*i*_ and *n*_*i*_ = *n*_*i*_^α^ + *n*_*i*_^β^ is the total occupation number of spatial orbital ϕ_*i*_.

## Computational Details

We consider several model closed-shell systems for which we can establish an exact benchmark using QED-FCI with a saturated photon basis, including LiH in a 6-311G basis set, H_2_O^2+^ cation, and BH_3_ in a 6-31G basis set. This selection of model systems includes a nonpolar (but polarizable) molecule (BH_3_), a polar molecule (LiH), and a molecule with net charge (H_2_O^2+^), which engenders a range of coupling behavior between the molecule and the photon field. After analyzing the behavior of both formulations of QED-CASCI for these three systems with respect to an exact benchmark, we conclude with an illustrative calculation of the naphthalene molecule treated in the cc-pVDZ basis set. Here, we explore the behavior of singlet polariton states for a range of coupling strengths leading to inversion of a singlet–triplet ordering in a (12,12) active space and also illustrate the scaling of our approach with respect to the number of photon states within this active space. For the LiH system, we scan a range of bond lengths to investigate ground and polariton surfaces. For the BH_3_ and C_10_H_8_ systems, we optimize the geometries using the second-order Møller-Plesset perturbation theory without cavity effects. The basis sets used in these optimizations for BH_3_ and C_10_H_8_ are 6-311G and cc-pVTZ, respectively. For the H_2_O^2+^ system, we use a typical geometry for neutral water. The ability to optimize geometries utilizing QED-CASCI nuclear forces will be the subject of future investigations. In all calculations, the exact ERIs are used; the only exception is the geometry optimization of C_10_H_8_ where the ERIs are approximated using density fitting. Our implementations makes use of the Psi4Numpy interface to obtain one- and two-electron integrals from the Psi4 quantum chemistry package.^85,86^ A link to a GitHub repository with our implementations and to a repository containing .json files with geometries and other parameters for all calculations is provided in the Data Availability section. Plots of orbitals for all active spaces considered in this work are provided in the Supporting Information in Figures S3–S6.

## Results and Discussion

Although we have remarked that the reference determinant in the coherent state basis includes an infinite number of photonic states by virtue of eq 6, this does not automatically guarantee that the photon basis for the subsequent QED-CASCI problem is complete. One reason for this is that the specific form of the coherent state transformation (i.e., the value of *z* in eq 3) is derived self-consistently from the QED-HF procedure, which is variationally optimized for the direct product of the coherent state wave function for the photon and a single Slater determinant for the electrons. Therefore, we can imagine that a different coherent state transformation exists for a given electronic state represented as a QED-CASCI or QED-FCI expansion. Nevertheless, we show that the coherent state formulation may provide a more rapidly converging photon basis for QED-CASCI approaches.

### LiH

**Figure 2:**
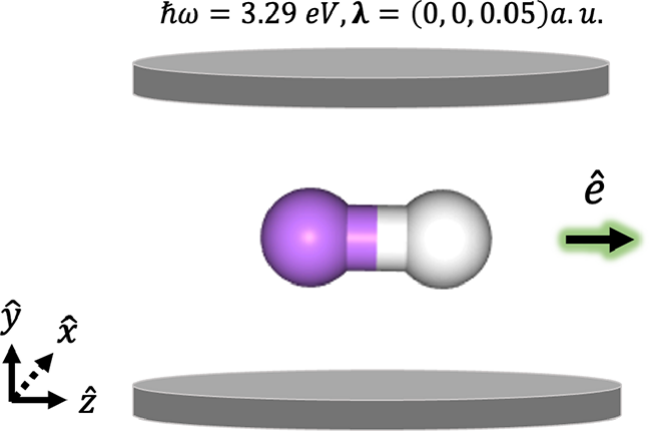
Schematic of the LiH system coupled to a cavity mode with **λ** = (0, 0, 0.05) a.u., polarized along the internuclear axis, and *ℏ**ω*** = 3.29 eV.

**Figure 3:**
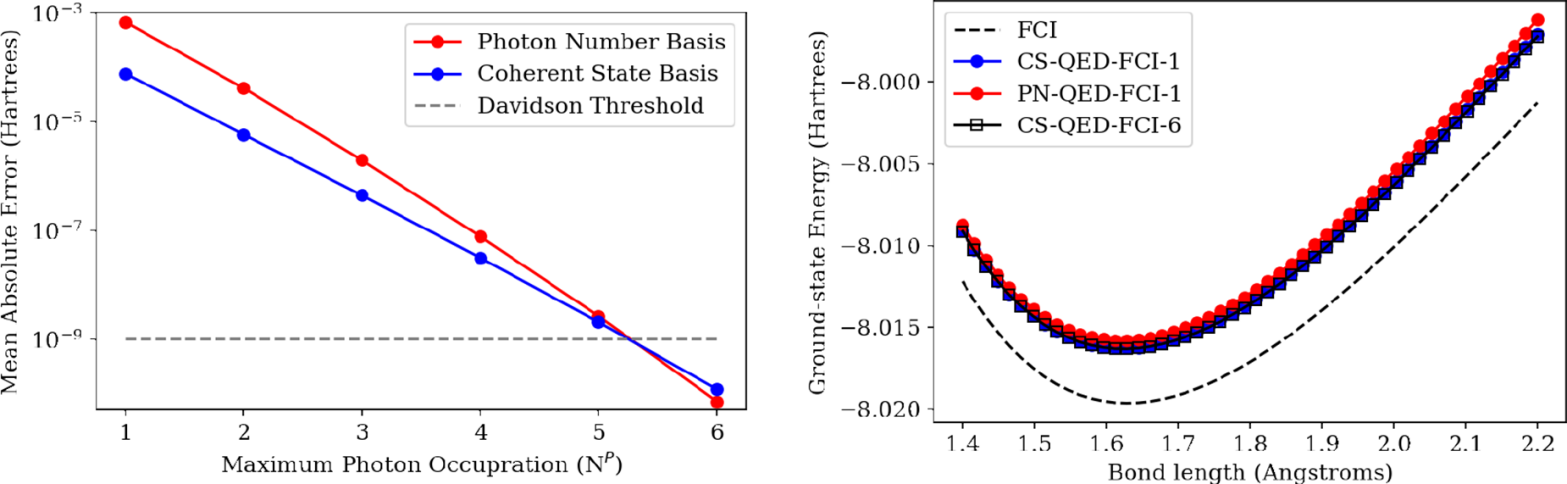
(Left) Mean absolute error across the ground-state potential energy scan of LiH coupled to a photon mode within the truncated Fock space with **λ** = (0, 0, 0.05) a.u. and *ℏ**ω*** = 3.29 eV as a function of the size of the photon basis in the photon number and coherent state representations. The reference energies come from CS-QED-FCI-10/6-311G; it can be seen that PN-QED-FCI-6 and CS-QED-FCI-6 are both fully photon-converged. (Right) The ground-state potential energy scan of LiH coupled to a photon mode within a truncated Fock space with **λ** = (0, 0, 0.05) a.u. and *ℏ**ω*** = 3.29 eV at the fully photon-converged CS-QED-FCI-6/6-311G level of theory compared to CS-QED-FCI-1/6-311G and PN-QED-FCI-1/6-311G, which represent minimal photon bases in the coherent state and photon number state formulations. The cavity-free FCI/6-311G energy is plotted for reference in the right panel.

To illustrate the convergence behavior of the photon basis, we first consider doing PN-QED-FCI and CS-QED-FCI on the bond stretch of the LiH diatomic molecule on a 6-311G basis. We consider this system coupled to a photon mode within a truncated Fock space with energy tuned to the lowest singlet excitation (*ℏ**ω*** = 3.29 eV) and with λ_*z*_ = 0.05 atomic units, polarized along the internuclear axis (see Figure 2). We choose this large value of the field to draw out a clear difference in the convergence behavior of the photon number and coherent state formulations. To explore this convergence behavior, the maximum photon occupation number is systematically increased from *N*^p^ = 1 to *N*^p^ = 10, where we could gauge that the photon space is fully converged by *N*^p^ = 6 since the further increase of *N*^p^ does not change the energies of any of the eigenvalues to within the convergence criteria of the Davidson solver (≈10^–9^ Hartrees). We examine the mean absolute error of the PES computed using PN-QED-FCI-*N*^p^ and CS-QED-FCI-*N*^p^ as a function of *N*^p^, and we see that this error is an order of magnitude smaller for the minimal photonic basis in the coherent-state formulation (see Figure 3 left panel). We see that the error decreases approximately linearly as a function of the size of the photonic basis for both formulations, with both approaching the convergence threshold of our Davidson solver by *N*^p^ ≈ 6 (see Figure 3 left panel). We use CS-QED-FCI-10 as the exact benchmark for the computation of the mean absolute errors displaced in the left panel of Figure 3. In the right panel of Figure 3, we show the ground-state potential energy stretch of the LiH both outside the cavity (dashed black line) and inside the cavity. For the latter, we compute the PES using PN-QED-FCI-1 and CS-QED-FCI-1 as a minimal photonic basis and CS-QED-FCI-6 as a fully converged photonic basis based on the behavior of the mean absolute error shown in the left panel of Figure 3. We see that all in-cavity PESs are above the cavity-free PES as expected. We also see CS-QED-FCI-6 is a lower bound to both curves in a minimal photonic basis, which follows from the variational nature of these calculations. Consistent with the idea that the coherent state formulation provides a more efficient photonic basis, we can see that the CS-QED-FCI-1 curve lies below the PN-QED-FCI-1 curve and is visually nearly identical with the fully converged result (Figure 3 right panel).

**Figure 4:**
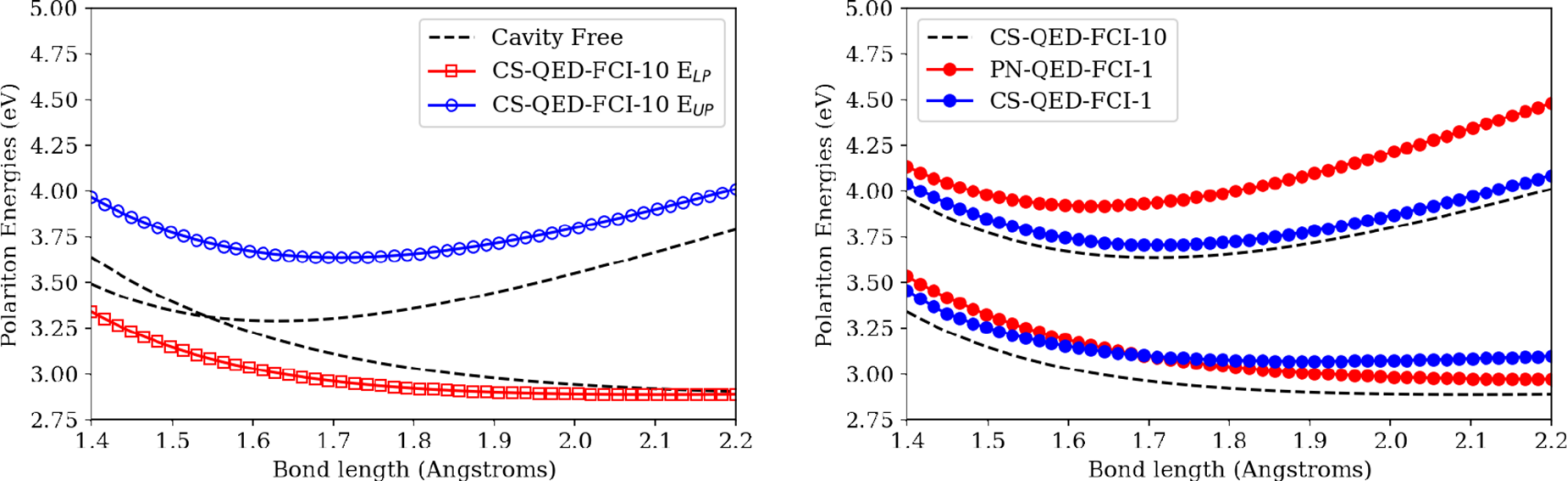
(Left) Polariton potential energy scans of LiH coupled to a photon mode within a truncated Fock space with **λ** = (0, 0, 0.05) a.u. and *ℏ**ω*** = 3.29 eV at the CS-QED-FCI-10/6-311G level as compared to the cavity-free FCI/6-311G energies of the ground-state surface displaced by *ℏ**ω*** and the first excited singlet state surface. (Right) The polariton potential energy surfaces in the minimal photon basis in the photon number representation (red) and the coherent state representation (blue) compared to the fully converged polariton potential energy surfaces.

Next, we consider the lower- and upper-polariton surfaces that emerge in the same LiH system. The left panel of Figure 4 shows the CS-QED-FCI-10/6-311G polariton surfaces (lower-polariton (LP) in red, upper-polariton (UP) in blue) along with the cavity-free FCI/6-311G surfaces for the ground-state displaced by the photon energy and the first singlet excited state potential energy surfaces (black dashed lines; see Figure 4 left panel). The magnitude of the field is evident by the large Rabi splitting between the LP and UP surfaces. We compare the LP and UP surfaces using a minimal photon basis (PN-QED-FCI-1 and CS-QED-FCI-1) to CS-QED-FCI-10 surfaces (Figure 4 right panel). We can see that the shapes of both polariton surfaces show sensitivity to the size of the photon basis in both the PN and CS formulations. However, the CS-QED-FCI-1 surfaces have greater parallelity with the photon-converged surfaces as compared to the PN-QED-FCI-1 surfaces. The coherent state transformation should shift the Hamiltonian to a frame where the bilinear coupling terms have a reduced magnitude, enabling faster convergence with the size of the photonic space. Just as with the ground-state surfaces for this system, we find that the polariton surfaces are fully photon-converged and are in numerical agreement with each other at the PN-QED-FCI-10 and CS-QED-FCI-10 levels (Figure S1 in the Supporting Information).

**Figure 5:**
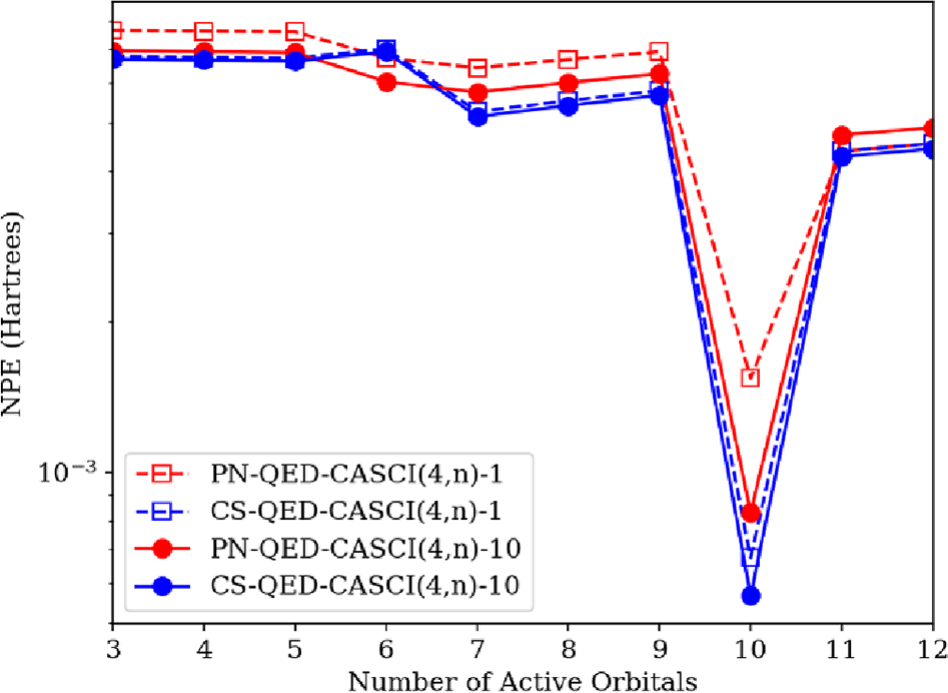
Nonparallelity error (NPE) of the ground-state potential energy surface of LiH coupled to a photon mode within the truncated Fock space with **λ** = (0, 0, 0.05) a.u. and *ℏ**ω*** = 3.29 eV at the QED-CASCI(4,*n*)-*N*^p^/6-311G level for *n* = [3, 12] active orbitals and *N*^*p*^ = 1 and 10. The NPE is computed relative to that of CS-QED-FCI-10/6-311G, which is fully photon-converged.

As a final analysis of the LiH system, we investigate the behavior of the ground-state potential energy surface as a function of the active space and the photonic basis size for the QED-CASCI method. Specifically, we compute the ground-state potential energy surfaces for the LiH cavity system at the CS-QED-CASCI(4,n)-*N*^p^/6-311G and PN-QED-CASCI(4,n)-*N*^p^/6-311G levels with *n* = [3, 12] and *N*^p^ = 1 and 10; note that a (4,16) active space is identical to QED-FCI for LiH in a 6-311G basis set. The nonparallelity errors (NPE) relative to CS-QED-FCI-10/6-311G are shown in Figure 5. Not surprisingly, the PN-QED-CASCI(4,n)-1 surfaces have the highest NPE for all active space sizes, while we observe CS-QED-CASCI(4,n)-10 surfaces having the lowest NPE for all active spaces except the (4,6) active space. Importantly, the CS-QED-CASCI(4,n)-1 NPE values are consistently lower than the PN-QED-CASCI(4,n)-10 results for all except the (4,6) active space as well. The larger NPE for the coherent state results in the (4,6) active space appears to arise from cavity modification to the orbitals that becomes pronounced when the bond is stretched. Evidently, in some contexts, the cavity Hartree–Fock procedure used to produce the orbitals for our CS implementation can degrade the quality of the orbital basis compared to that of the canonical Hartree–Fock orbitals. In Figure S2, we show the ground-state surfaces from which the (4,6) NPEs were derived, where we observe the CS methods are nearly identical to the PN-QED-CASCI(4,6)-10 curve at short bond lengths and then rise above this curve at longer bond lengths. In Figure S3, we show plots of the orbitals that are used as the basis of the PN- and CS- calculations at representative short (*r* = 1.4 Å) and long (*r* = 1.9 Å) bond lengths. Occupation numbers for several active space sizes and for the full CI limit are provided in Table S1. We see that orbital 6 is particularly affected by the cavity at longer bond lengths, which is likely the origin of the change in NPE behavior that we observe in the (4,6) active space. Because we use canonical Hartree–Fock orbitals in our PN formulation, this effect is not observed in the PN results. This suggests that there can be additional sensitivity to the size of the photon basis when the electronic excitation space is truncated, which was not evident in the QED-FCI results presented in Figure 3.

### H_2_O^2+^

Next, we consider a model charged system, H_2_O^2+^, in the 6-31G basis set coupled to a photon mode within a truncated Fock space with *ℏ**ω*** = 10 eV with a polarization vector **λ** = (0, 0, 0.01) in atomic units. There are no optically allowed transitions in the UV–vis region for this system; the photon energy *ℏ**ω*** is not resonant with any transitions from the FCI/6-31G eigenspectra in this species and is arbitrarily chosen; consequently, the dipole self-energy dominates the coupling behavior. We keep the geometry fixed at *r*_*OH*_ = 1.0 Å and θ_*HOH*_ = 104.5°, which is a typical geometry for neutral water. While this will surely not correspond to an equilibrium geometry for H_2_O^2+^, we simply seek to study the behavior of the QED-CASCI method for a charged closed-shell system for which we can provide a QED-FCI benchmark. Plots of the canonical RHF and QED-RHF orbitals for this system are shown in Figure S4.

Charged molecular species, unlike their neutral counterparts, have origin-dependent dipole moments. A demonstration following ref (87) follows. Consider the dipole operator for a molecular species **μ̂** = ∑_*i*_*Z*_*i*_**r**_*i*_ where *Z*_*i*_ and **r**_*i*_ are the charge and position operator/coordinate for the *i*^th^ particle. Shifting the molecule by Δ**r** gives a displaced operator as follows, **μ̂***′* = ∑_*i*_*Z*_*i*_(**r**_*i*_ + Δ**r**) = ∑_*i*_*Z*_*i*_**r**_*i*_ + Δ**r**∑_*i*_*Z*_*i*_. The operator **μ̂***′* is identical to **μ̂** for neutral species where the sum of charges ∑_*i*_*Z*_*i*_ is zero, whereas it is clearly changed for charged species where this sum is nonzero. This shift in position for the molecule can be formulated in terms of a Unitary transformation *Û* = *e*^*i*Δ**r***p̂*^ where *p̂* is the momentum operator for the molecular degrees of freedom, so that we can view the Pauli-Fierz Hamiltonian for any molecule shifted from the origin as *Û*^†^*Ĥ*_PF_*Û*.^87^ Because Unitary transformations are eigenvalue preserving, we know that the energies associated with neutral and charged species must be origin invariant. However, it is important to note that we are only guaranteed to recover these origin-invariant energies in the limit in which we can find the exact eigenvalues of our shifted Hamiltonian. There is discussion of several approximate *ab initio* QED methods, including QED-HF and QED-CC with incomplete photon number bases, that suffer from origin-dependent errors for charged species.^33,48^

**Figure 6:**
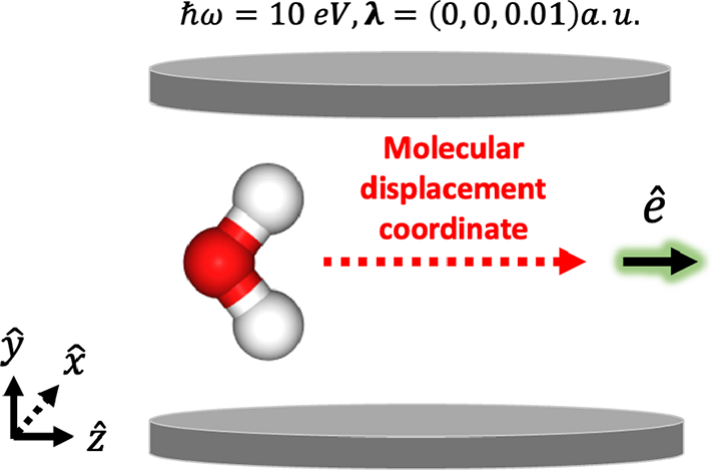
Schematic of H_2_O^2+^ system coupled to a cavity mode with **λ** = (0, 0, 0.01) a.u. and *ℏ**ω*** = 10 eV where the origin of the molecule is systematically displaced along the polarization direction.

**Figure 7:**
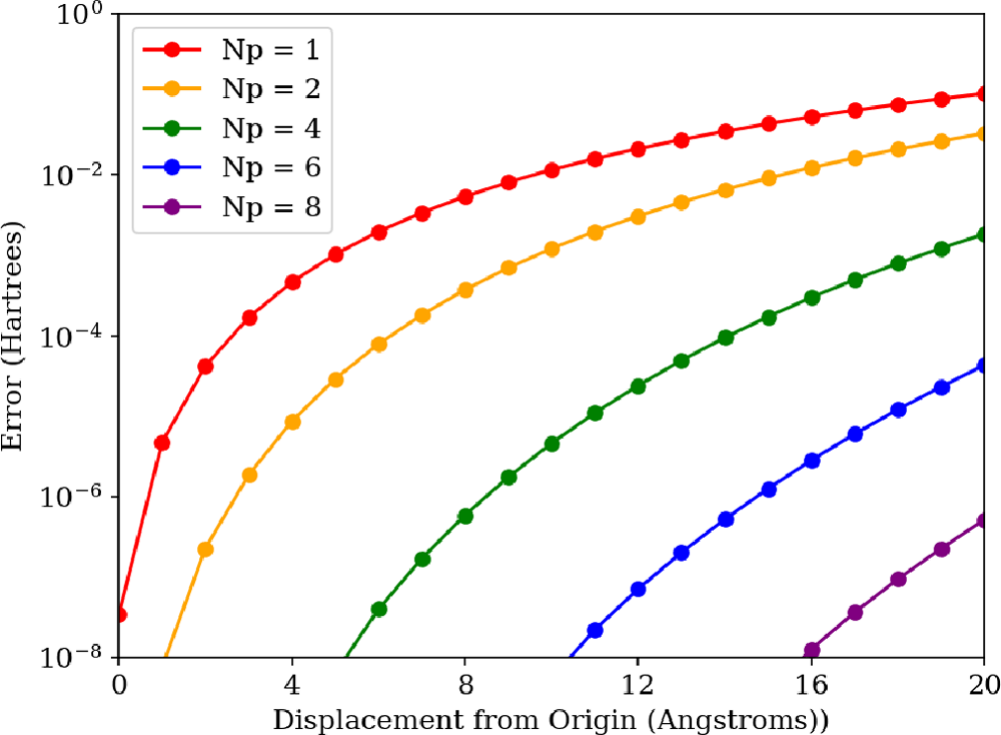
Energy error of the H_2_O^2+^ lowest-energy singlet state coupled to a photon mode within truncated Fock space with **λ** = (0, 0, 0.01) a.u. and *ℏ**ω*** = 10 eV as a function of displacement from the origin for different sizes of the photon basis for the photon number formulation of PN-QED-FCI-*N*^p^/6-31G; here *N*^p^ = 1 corresponds to the minimal photon basis including the |0^p^⟩ and |1^p^⟩ occupation states. The reference energies for each displacement come from CS-QED-FCI-1/6-31G, which is origin invariant.

The origin-dependence of QED-HF can be completely alleviated through saturation of the photon basis set or, alternatively, through formulating QED-HF in the coherent-state basis which can also be viewed as a position shift for the photon coordinate that compensates for the displacement of the charged particles.^33^ Similarly, we observe that origin-invariant energies are obtained from the CS-QED-FCI-1 approach. However, the PN-QED-FCI-*N*^p^ approach does not generally produce origin-invariant energies for charged species. We examine the energy error in the lowest-energy singlet state of H_2_O^2+^ computed by PN-QED-FCI-*N*^p^ while displacing the center of mass along the *z*-axis (see Figure 6), which is the polarization direction of the field and the direction along which the dipole moment of H_2_O^2+^ is oriented. We see that when *N*^*p*^ = 1 (the minimal photon basis, including |0^p^⟩ and |1^p^⟩ photon number states), the energy error approaches the milliHartree range by a displacement of 4 Å from the origin and continues to increase with further displacement (see Figure 7). We also clearly see the mitigating impact that growing the size of the photonic basis has on the origin dependence; for *N*^p^ = 8, the error is in the microHartree range after a displacement of 20 Å (see Figure 7). We emphasize that since we are performing full CI within the electronic basis, these origin-dependent energy errors arise solely from truncation of the photonic basis. As we approach the completeness of the photonic basis, we have a fully variational solution within the single electron orbital representation given by the 6-31G basis set, and see that the origin invariance of the energies is properly restored (see Figure 7).

**Figure 8:**
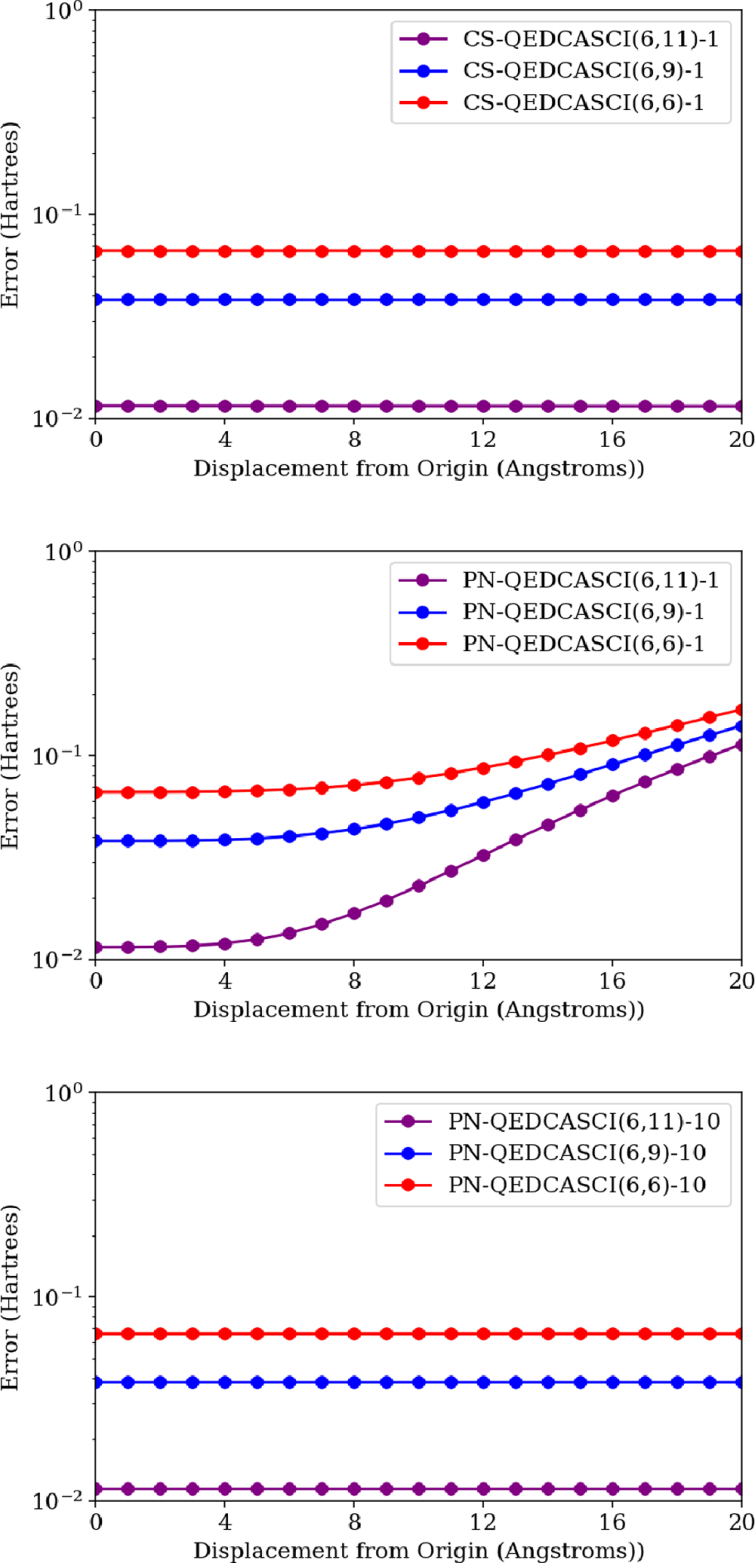
Energy error of the H_2_O^2+^ lowest-energy singlet state coupled to a photon mode within a truncated Fock space with **λ** = (0, 0, 0.01) a.u. and *ℏ**ω*** = 10 eV as a function of displacement from the origin for CS-QED-CASCI(6,n)-1 (top), PN-QED-CASCI(6,n)-1 (middle), and PN-QED-CASCI(6,n)-10 (bottom) for *n* = 11, 9, 6 all in a 6-31G basis set. The coherent state formulation shows negligible origin dependence as compared to the correlation error arising from the truncated active space, and the photon number formulation shows strong origin dependence when *N*^*p*^ = 1 and negligible origin dependence when *N*^*p*^ = 10.

A logical next question is how the QED-CASCI methods perform under truncation of the photonic basis since here we cannot achieve a fully variational solution even with saturation of the photonic subspace. That is, in QED-CASCI just as in CASCI, the CI coefficients in eq 7 are variationally optimized, but the orbital basis is not; this is to be contrasted with approaches like CASSCF/MCSCF, which is fully variational in the sense that the CI coefficients and the orbital basis are variationally optimized.^34^ The behavior of QED-CASSCF as a fully variational approach with cavity effects will be a topic of future investigation. We explore this question using three different active spaces: (6,11), (6,9), and (6,6). All active spaces have excluded 2 core electrons, and the (6,11) active space excludes the two highest virtual orbitals; i.e., an (8,13) active space is equivalent to FCI for the electronic subspace of this system. We observe that CS-QED-CASCI-1 is nearly origin-independent; in the singlet ground-state energy the subtle origin dependence is negligible compared to the correlation energy that is neglected by truncation of the excitation space (see Figure 8 Top Panel and Table 1). We conjecture this slight origin dependence arises because the specific form of coherent state transformation used (specifically the parameter *z* in eq 3) derives from the QED-HF reference wave function and has some error relative to a coherent state transformation that would be derived from a QED-CASCI state. By comparison, the PN-QED-CASCI-1 shows strong origin dependence that becomes appreciable compared to the correlation error at displacements greater than or equal to 8 Å (see Figure 8 middle panel and Table 1). Again, we see that expanding the size of the photon basis to *N*^*p*^ = 10 alleviates the origin dependence, giving results that are independent of origin (see Figure 8 bottom panel and Table 1).

**Table 1:** Absolute Energy Error of the Lowest Energy Singlet State of H_2_O^2+^ Displaced by 20 Å Relative to the Origin CS-QED-CASCI(6,n)-1, PN-QED-CASCI(6,n)-1, and PN-QED-CASCI(6,n)-10 Levels of Theory, All in the 6-31G Basis Set

Absolute Error at Maximum Displacement (Hartrees)			
	(6,11)	(6,9)	(6,6)
CS *N*^*p*^ = 1	4.90 × 10^–5^	6.62 × 10^–5^	7.62 × 10^–5^
PN *N*^*p*^ = 1	1.01 × 10^–1^	1.01 × 10^–1^	1.02 × 10^–1^
PN *N*^*p*^ = 10	3.84 × 10^–9^	3.82 × 10^–9^	3.85 × 10^–9^

### BH_3_

**Figure 9:**
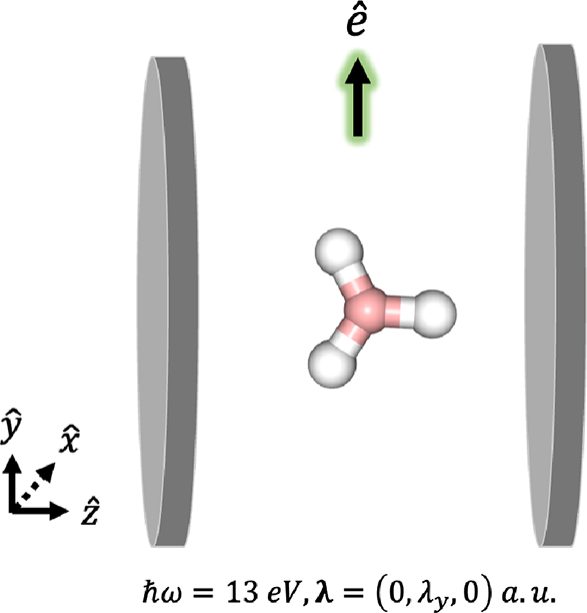
Schematic of BH_3_ system coupled to a cavity mode with variable **λ** polarized along the *y*-axis and *ℏ**ω*** = 13 eV.

**Figure 10:**
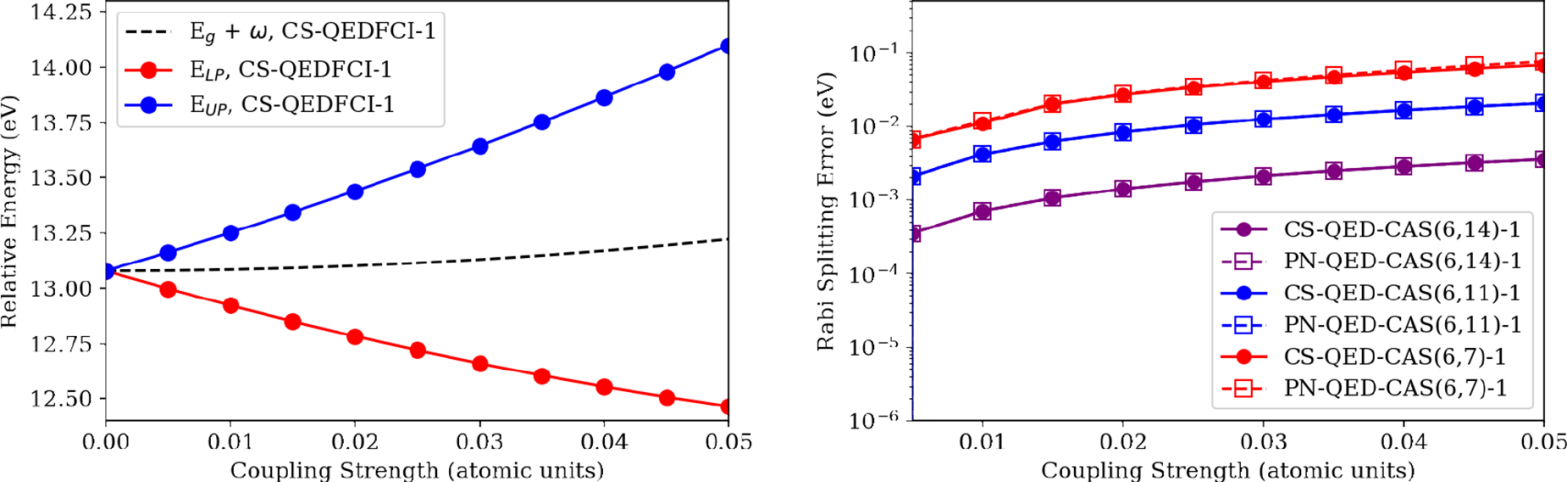
(Left) CS-QED-FCI-1/6-31G in the coherent state basis of BH3 coupled to a photon mode within a truncated Fock space with *y*-polarization tuned to a transition with a strong *y*-component of the transition dipole moment (third singlet excited state); the geometry is optimized at the MP2/6-311G level of theory. Photon energy: 13.07 eV. (Right) Rabi splitting using QED-CASCI(n,m)/6-31G in the coherent state basis of BH_3_ coupled to a photon with *y*-polarization tuned to a transition with a strong *y*-component of the transition dipole moment (third singlet excited state); the photon energy geometry is optimized at the MP2/6-311G level of theory. Photon energy is 13.65 eV for CASCI(6,7), 13.25 eV for CASCI(6,11), and 13.09 eV for CASCI(6,14).

As a final system with a QED-FCI benchmark, we consider BH_3_ within the 6-31G basis set as a nonpolar model system. At the FCI/6-31G level, the third singlet excited state with an excitation energy of 13.07 eV has a strong transition dipole moment oriented along the *y*-axis in the coordinate system illustrated in Figure 9. We first compute the polariton energies at the CS-QED-FCI-1/6-31G level (see Figure 10) for a range of coupling strengths up to λ_*y*_ = 0.05 a.u. We then computed the polariton energies at the same coupling strengths at the QED-CASCI-1(6,14)/6-31G, QED-CASCI-1(6,11)/6-31G, and QED-CASCI-1(6,7)/6-31G in both photon number and coherent state representations. In each case, we used photon energies tuned to the analogous optically allowed transition at the corresponding CASCI(*N*_el_, *N*_orb_)/6-31G level of theory: *ℏ**ω*** = 13.65 eV for CASCI(6,7)/6-31G, *ℏ**ω*** = 13.25 eV for CASCI(6,11)/6-31G, and *ℏ**ω*** = 13.09 eV for CASCI(6,14)/6-31G. As all of these levels of theory will result in different absolute energies of the polariton states, we compare the Rabi splitting energy (defined as the difference between the upper polariton and lower polariton energies) at each level of theory to the CS-QED-FCI-1/6-31G Rabi splitting for all values of λ_*y*_ > 0 (see Figure 10 right panel); the Rabi splitting error goes to 0 by definition with λ_*y*_ = 0. In this system, we see very little difference between the computed Rabi splitting in the photon number and coherent state representations for a given QED-CASCI active space size (Figure 10 right panel). However, we do see that the error in the Rabi splitting is rather sensitive to the size of the active space, with the largest (6,14) active space having consistently the smallest error in the Rabi splitting, with errors around 1 meV/36 microHartrees at the strongest coupling strength (λ_*y*_ = 0.05 a.u.). By contrast, the smallest active space (6,7) approaches errors in the Rabi splitting of around 0.1 eV/3.6 mHartree at the strongest coupling strength. The strong dependence of the Rabi splitting error on the active space size suggests that the inclusion of many-body correlation effects along with electron–photon coupling can impact the ability to capture the essential phenomenology of molecular polaritons.

### C_10_H_8_

**Figure 11:**
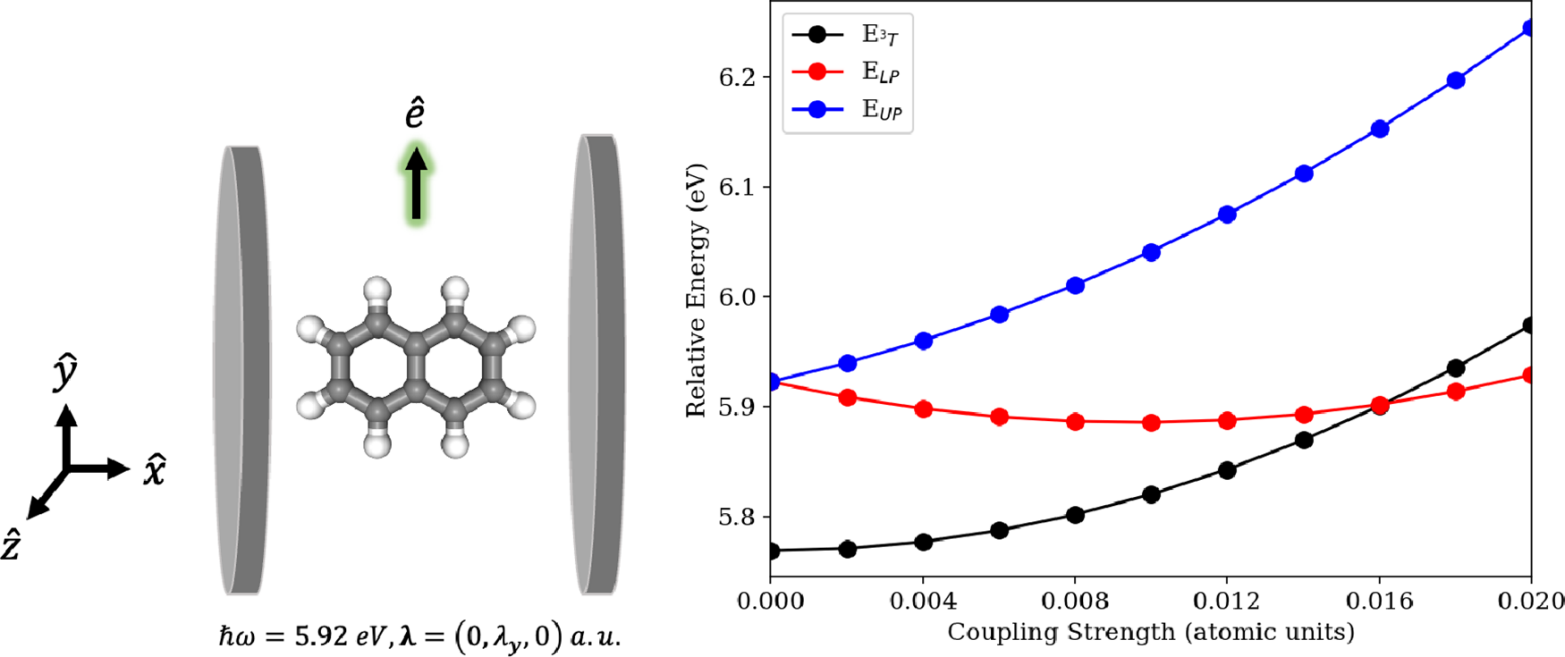
(Left) Schematic of the C_10_H_8_ system coupled to a photon mode within a truncated Fock space with variable **λ** polarized along the *y*-axis and *ℏ**ω*** = 5.92 eV. (Right) Relative energies of the polariton states emerging from coupling the ^0^S → ^2^S transition to the cavity photon with *ℏ**ω*** = 5.92 eV plotted along with the relative energy of a nearby triplet state ^3^T. The energies are computed relative to the ground-state energy of naphthalene under zero coupling computed at the CASCI(12,12)/cc-pVDZ level.

Our final system of study is the naphthalene molecule (C_10_H_8_) coupled to a photon mode within a truncated Fock space tuned to the transition between the ground state and the second singlet excited state (^0^S and ^2^S). This represents the simplest embodiment of the polycyclic aromatic hydrocarbons (PAH) family of molecules that are paradigmatic molecular systems for strong multireference correlation effects.^70,88−91^ Cavity effects in aggregates of PAH systems have also been proposed as a route to enhance singlet fission^92^ and inversion of singlet–triplet gaps.^93^ Here we investigate the ordering of singlet polariton states relative to a nearby triplet state using the CS-QED-CASCI approach. In future work, we will pursue some of the aforementioned approaches that enable large active spaces so that cavity effects in a broader class of PAH systems may be studied.^66−69^ At the optimized geometry of C_10_H_8_, we find that the second singlet excited state at both the CASCI(10,10)/cc-pVDZ and CASCI(12,12)/cc-pVDZ levels has a transition energy of 5.92 eV and a strong transition dipole moment along the *y*-axis. The (10,10) active space includes all electrons and orbitals comprising the aromatic system; however, we find that the evolution of the polariton energies becomes nonsmooth for values of λ_*y*_ > 0.01 atomic units in this active space, while we observe smooth evolution of the energies for all values of λ_*y*_ considered when a (12,12) active space is used. In Figure S6, we show plots of all active orbitals with and without cavity effects and observe that the cavity effects lead to some reordering of the high-lying virtual orbitals. Expanding the active space to (12,12) enables a more consistent set of orbitals in the active space across the range of coupling strengths considered. We track the evolution of the polariton state energies that emerge from coupling the ground-to-second singlet excited-state (^0^S → ^2^S) transition along with the energy of a nearby triplet state, which is the third triple excited state (here denoted ^3^T). We can see that outside the cavity the energy of the ^2^S state lies approximately 0.16 eV above the energy of the ^3^T state (see Figure 11 right panel under zero coupling). Increasing the coupling strength monotonically increases the upper polariton energy and the energy of the ^3^T state. While the lower polariton energy initially decreases with increasing coupling strength, we see that it starts to increase for values of λ_*y*_ ≈ 0.012 atomic units. Nevertheless, we see that the ordering of the ^3^T and singlet lower polariton state changes at around λ_*y*_ = 0.016 atomic units. Such an inversion of singlet and triplet states was reported in TADF materials by Kéna-Cohen and co-workers^93^ in the collective strong coupling regime. Here we wish to point out a somewhat subtle point of distinction between the behavior of these states in the single molecule coupling regime discussed in this work and the collective coupling regime discussed in ref (93). Namely, in the collective coupling regime, the Hamiltonian contribution we call *Ĥ*_blc_ would experience a scaling with the number of molecules coupled to a mode, whereas the term *Ĥ*_dse_ would not experience this scaling, and this term will affect the Rabi splitting between the singlet polariton states but will not affect the energetics of the triplet states due to the absence of a transition dipole moment. However, the increase in the triplet energy we observe arises from the dipole self-energy term (which can couple to the light field through molecular dipole and quadrupole terms), which does not experience number scaling in the collective coupling regime. Hence, the triplet energy is not observably modified under collective strong coupling.^93^

We also report on the performance of the QED-CASCI method using a naphthalene system. Specifically, we monitor the total number of determinants comprising eq 7, the memory size of the **σ** and CI vectors, and the total time required to converge eigenstates for a series of CS-QED-CASCI(12,12)-*N*^p^/cc-pVDZ calculations. For each calculations, we set *ℏ**ω*** = 5.92 eV, **λ** = (0, 0.01, 0) a.u., and solve for the 5 lowest roots of the Hamiltonian. As expected, the number of determinants increases linearly with the size of the photonic basis, specifically as (*N*^p^ + 1) × *N*_det_^e^ where *N*_det_^e^ is the number of determinants in the electronic subspace that is determined by a given active space size^63,64^ (see Table 2). The sizes of **σ** and CI vectors are calculated for the iteration when the maximum size of the subspace is reached during the Davidson iterations; we set the threshold for this maximum value to be 12 for all calculations reported in Table 2, which limits the total size of these vectors to be proportional to 12 × 5 × (*N*_*p*_ + 1) × *N*_*det*_, where 5 again comes from the number of roots being solved for. The time to solution tends to be close to linear with the total number of determinants, with the longest time to solution of <4 h seen for the *N*^p^ = 100 case (see Table 2).

**Table 2:** Timings of the Davidson Iterative Process for CS-QED-CASCI(12,12)-*N*^p^/cc-pVDZ for Different Values of *N*^p^^a^

*N*^p^	Number of Determinants	Total size of **σ** and CI vectors (MB)	Time (s)
1	1707552	1563.4	210.0
2	2561328	2345.0	340.4
4	4268880	3908.2	607.9
10	9391536	8598.2	1429.6
20	17929296	16414.8	2667.9
40	35004816	32047.8	5261.0
100	86231376	78947.2	12864.9

a Timings were performed on a Dell Precision 7920 running Ubuntu 22 with a single 3.9 GHz Intel Xeon Gold 6250 processor.

## Concluding Remarks

We developed an approach called QED-CASCI to provide ground- and excited-state polaritonic surfaces with a balanced description of strong correlation effects among electronic and photonic degrees of freedom. This method can provide a platform for ai-CQED when both strong electron correlation and strong light–matter coupling are important, with one example being the cavity-enhanced isomerization of the azobenzene.^94^ Application of this method to the simulation of chemical transformations will require forces and couplings from multiple polaritonic potential energy surfaces, and future work will focus on the development of technology to provide these quantities. We have implemented two different formulations of QED-CASCI: PN-QED-CASCI which is formulated on the photon-number basis and CS-QED-CASCI which is formulated on the coherent state basis. Both methods were applied to a range of model systems for which we can also provide a numerically exact benchmark using QED-FCI. We have shown that both methods converge to numerically identical answers in the limit that the photon basis becomes complete but that CS-QED-CASCI shows accelerated convergence that becomes particularly prominent for polar and charged species. The efficiency of our serial implementation was demonstrated on the naphthalene molecule in a (12,12) active space where we can solve for multiple polaritonic states with the inclusion of 100 photonic basis states in roughly 4 h on a single 8-core CPU.

## Data Availability

The implementation of the QED-CASCI method used for the results presented within can be accessed in the following GitHub repository: https://github.com/mapol-chem/qed-ci/tree/jctc_submission. The data that support the findings of this study are available from the corresponding author upon reasonable request and are openly available in GitHub at https://github.com/FoleyLab/data_repository/tree/jctc_submission/Mapol/. For LiH data: https://github.com/FoleyLab/data_repository/tree/jctc_submission/Mapol/LiH/. For H_2_O^2+^ data: https://github.com/FoleyLab/data_repository/tree/jctc_submission/Mapol/H2O_ions/. For BH_3_ data: https://github.com/FoleyLab/data_repository/tree/jctc_submission/Mapol/BH3/. For C_10_H_8_ data: https://github.com/FoleyLab/data_repository/tree/jctc_submission/Mapol/Napthalene/.

## Supporting Information Available

Additional surface plots for the LiH system, occupation numbers for QED-CASCI and QED-FCI calculations on the LiH system, orbital plots for all systems, and a representative algorithm for the construction of modified σ vector terms (PDF)
