# Supplementary material for: Cavity Quantum Electrodynamics Complete Active Space Configuration Interaction Theory
Source: J Chem Theory Comput. 2024 Jan 30;20(3):1214–27. doi: 10.1021/acs.jctc.3c01207 (PMC10876286; doi:10.1021/acs.jctc.3c01207)
Supplement: Supplementary file 1 — ct3c01207_si_001.pdf [file ct3c01207_si_001.pdf]

Supporting Information for Cavity Quantum Electrodynamics Complete Active Space Configuration Interaction Theory

Nam Vu,*,† Daniel Mejia Rodriguez, Nicholas P. Bauman, Ajay Panyala, Erdal Mutlu,‡ Niranjan Govind,‡,¶ and Jonathan J. Foley IV*,†

†*Department of Chemistry, University of North Carolina Charlotte, 9201 University City Blvd, Charlotte, North Carolina 07470A*

‡*Physical and Computational Sciences Directorate, Pacific Northwest National Laboratory, Richland, WA 99352*

¶*Department of Chemistry, University of Washington, Seattle, WA 98195, USA*

E-mail: nvu12@charlotte.edu; jfoley19@charlotte.edu

Additional Surface Plots

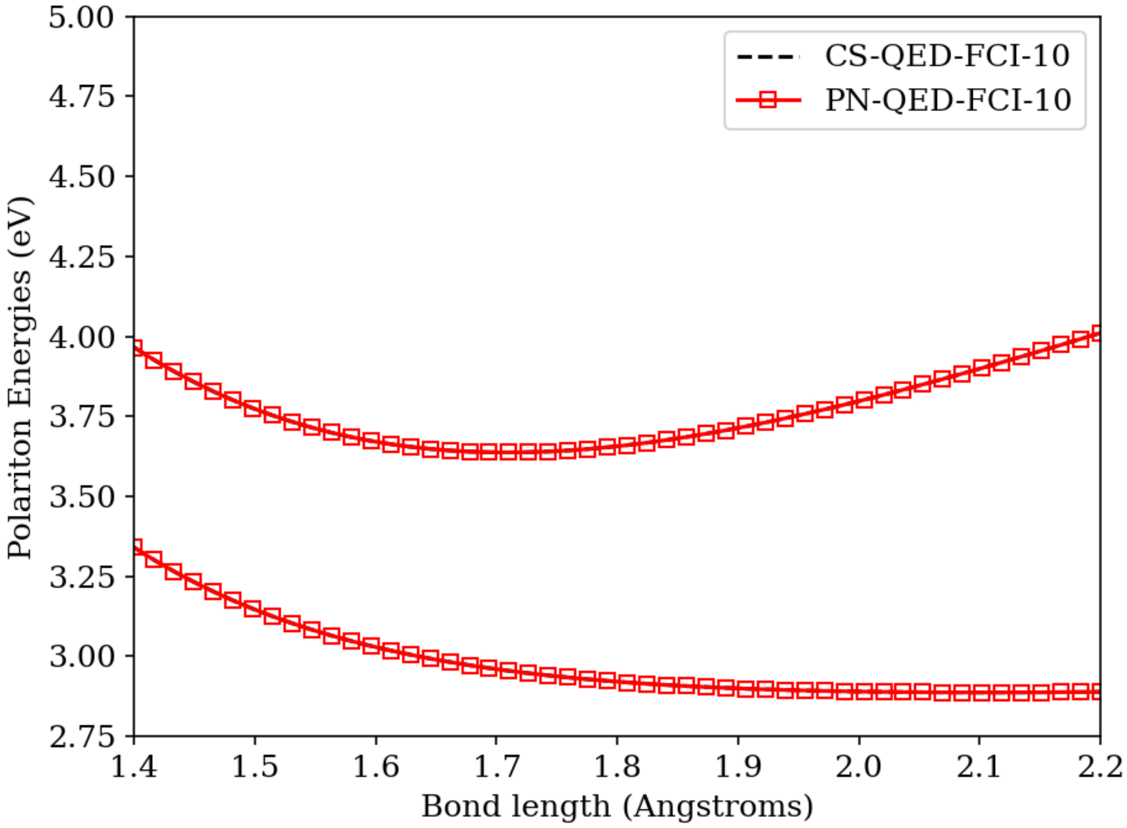

Figure S1: The polariton potential energy scans of LiH coupled to a photon with $\boldsymbol{\lambda} = (0, 0, 0.05)$ a.u. and $\hbar\omega = 3.29$ eV at the CS-QED-FCI-10/6-311G level and the PN-QED-FCI-10/6-311G level.

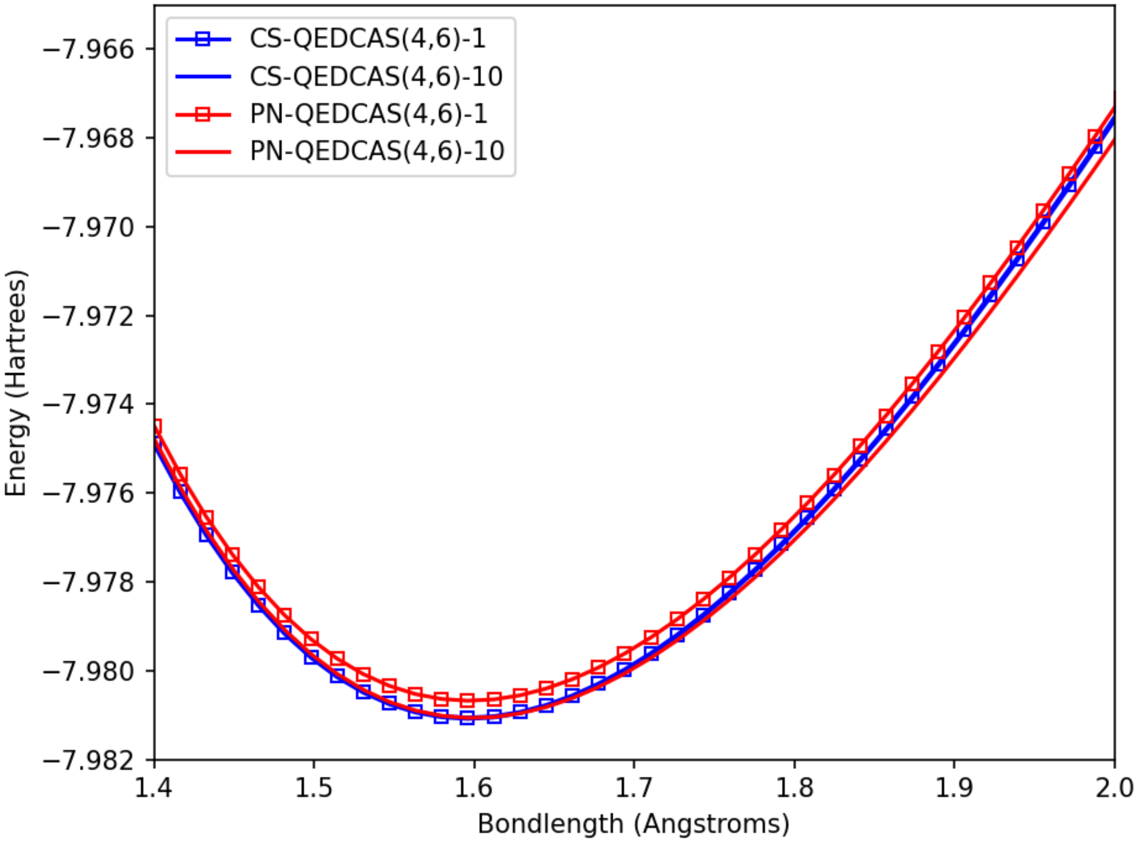

Figure S2: The polariton potential energy scans of LiH coupled to a photon with $\boldsymbol{\lambda} = (0, 0, 0.05)$ a.u. and $\hbar\omega = 3.29$ eV at the CS-QED-FCI-10/6-311G level and the PN-QED-FCI-10/6-311G level.

Select Orbital Plots and Occupation Numbers

Table S1: Occupation Numbers for different active spaces in the photon-number (PN) and coherent-state (CS) representations 10 photonic states for LiH with a bond length of 1.4 Angstroms coupled to a photon with $\lambda_z = 0.05$ atomic units and $\hbar\omega = 3.29$ eV

Orbital Number	PN-(4,4)	CS-(4,4)	PN-(4,6)	CS-(4,6)	PN-FCI	CS-FCI
1	2.0000	2.0000	2.0000	2.0000	1.9969	1.9969
2	1.9992	1.9987	1.9975	1.9968	1.9555	1.9544
3	0.0007	0.0012	0.0013	0.0019	0.0067	0.0097
4	0.0001	0.0001	0.0001	0.0001	0.0003	0.0004
5	0.0000	0.0000	0.0001	0.0001	0.0003	0.0004
6	0.0000	0.0000	0.0011	0.0011	0.0070	0.0052
7	0.0000	0.0000	0.0000	0.0000	0.0012	0.0028
8	0.0000	0.0000	0.0000	0.0000	0.0009	0.0008
9	0.0000	0.0000	0.0000	0.0000	0.0009	0.0008
10	0.0000	0.0000	0.0000	0.0000	0.0092	0.0078
11	0.0000	0.0000	0.0000	0.0000	0.0079	0.0075
12	0.0000	0.0000	0.0000	0.0000	0.0003	0.0002
13	0.0000	0.0000	0.0000	0.0000	0.0003	0.0002
14	0.0000	0.0000	0.0000	0.0000	0.0094	0.0095
15	0.0000	0.0000	0.0000	0.0000	0.0011	0.0011
16	0.0000	0.0000	0.0000	0.0000	0.0024	0.0024

Table S2: Occupation Numbers for different active spaces in the photon-number (PN) and coherent-state (CS) representations 10 photonic states for LiH with a bond length of 1.9 Angstroms coupled to a photon with $\lambda_z = 0.05$ atomic units and $\hbar\omega = 3.29$ eV

Orbital Number	PN-(4,4)	CS-(4,4)	PN-(4,6)	CS-(4,6)	PN-FCI	CS-FCI
1	2.0000	2.0000	2.0000	2.0000	1.9969	1.9969
2	1.9991	1.9984	1.9971	1.9965	1.9530	1.9518
3	0.0008	0.0014	0.0015	0.0022	0.0075	0.0107
4	0.0001	0.0001	0.0001	0.0001	0.0003	0.0003
5	0.0000	0.0000	0.0001	0.0001	0.0003	0.0003
6	0.0000	0.0000	0.0012	0.0011	0.0074	0.0050
7	0.0000	0.0000	0.0000	0.0000	0.0012	0.0033
8	0.0000	0.0000	0.0000	0.0000	0.0008	0.0007
9	0.0000	0.0000	0.0000	0.0000	0.0008	0.0007
10	0.0000	0.0000	0.0000	0.0000	0.0102	0.0087
11	0.0000	0.0000	0.0000	0.0000	0.0075	0.0071
12	0.0000	0.0000	0.0000	0.0000	0.0002	0.0002
13	0.0000	0.0000	0.0000	0.0000	0.0002	0.0002
14	0.0000	0.0000	0.0000	0.0000	0.0002	0.0104
15	0.0000	0.0000	0.0000	0.0000	0.0011	0.0011
16	0.0000	0.0000	0.0000	0.0000	0.0024	0.0011

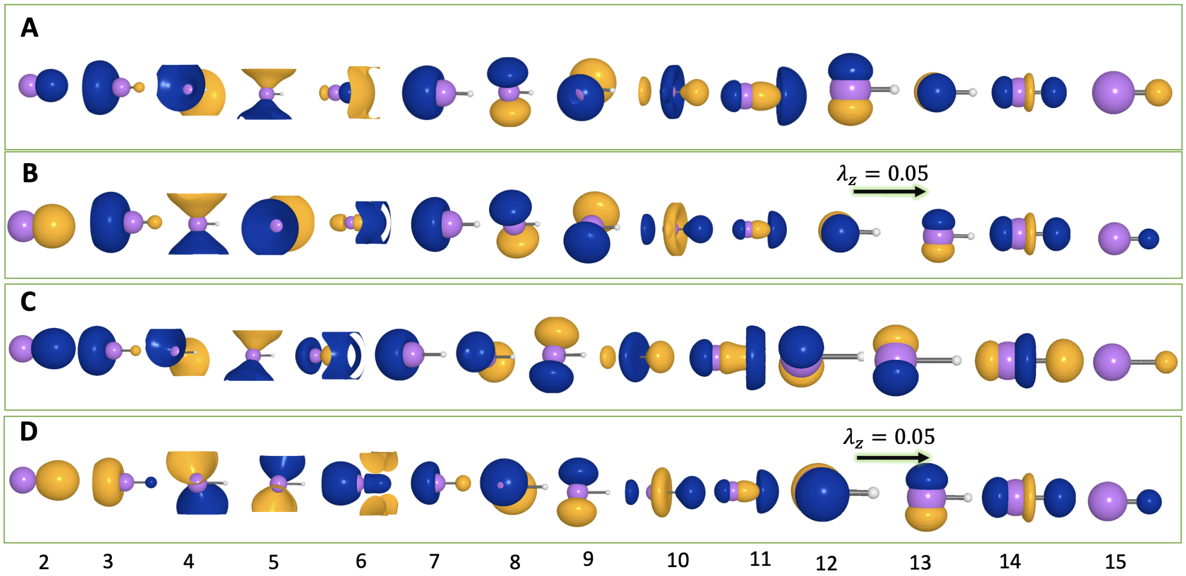

Figure S3: (A) RHF/6-311G Orbitals for LiH with a bondlength of 1.4 Angstroms. (B) QED-RHF/6-311G Orbitals for LiH with a bondlength of 1.4 Angstroms coupled to a field with $\lambda_z = 0.05$ atomic units. (C) RHF/6-311G Orbitals for LiH with a bondlength of 1.9 Angstroms. (D) QED-RHF/6-311G Orbitals for LiH with a bondlength of 1.9 Angstroms coupled to a field with $\lambda_z = 0.05$ atomic units.

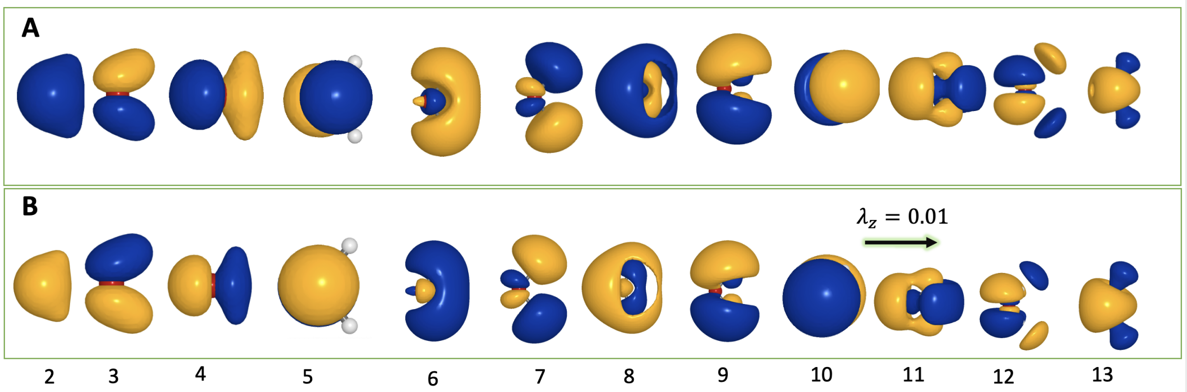

Figure S4: (A) RHF/6-31G Orbitals for H_2O^{2+} system at the origin. (B) QED-RHF/6-31G Orbitals for H_2O^{2+} system coupled to a cavity mode with $\boldsymbol{\lambda} = (0, 0, 0.01)$ a.u. at the origin.

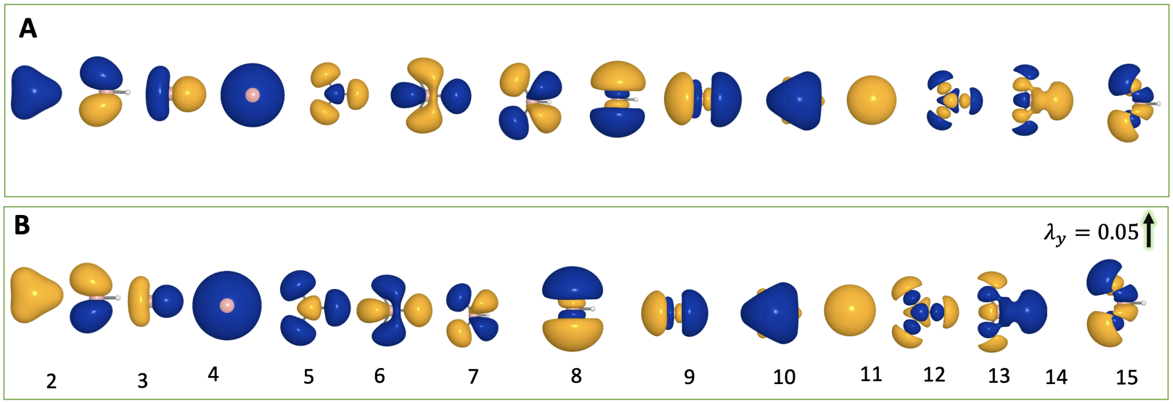

Figure S5: (A) RHF/6-31G Orbitals for BH_3 system. (B) QED-RHF/6-31G Orbitals for BH_3 system coupled to a cavity mode with $\boldsymbol{\lambda} = (0, 0.05, 0.0)$ a.u.

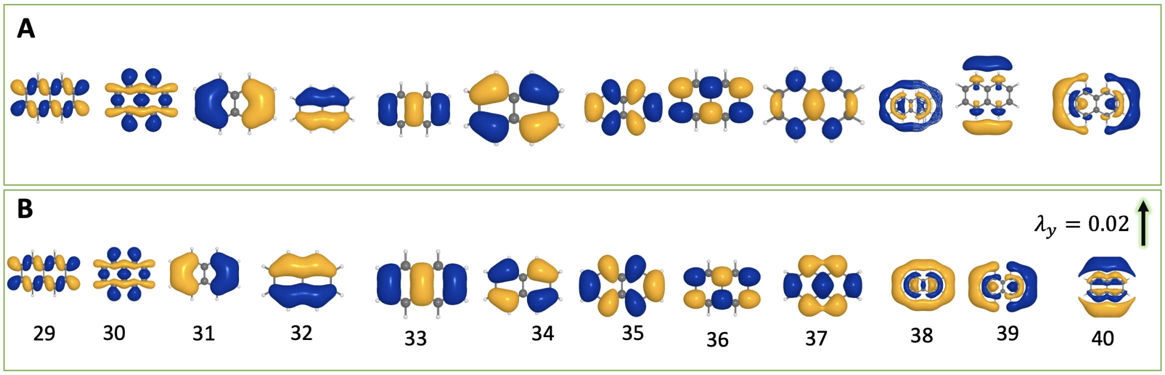

Figure S6: (A) RHF/cc-pVDZ Orbitals for $C_{10}H_8$ system. (B) QED-RHF/cc-pVDZ Orbitals for$C_{10}H_8$ system coupled to a cavity mode with $\boldsymbol{\lambda} = (0, 0.02, 0.0)$ a.u.

Algorithm for building the second term in the σ_3 vector

Algorithm S1 build the second term of sigma 3

procedure BUILD_SIGMA3_2
  for $m \leftarrow 0,$ number of photon $- 1$ **do**
    for $I_\beta \leftarrow 0,$ number of beta strings $- 1$ **do**
      $F \leftarrow 0$
      for $j \leftarrow 0,$ number of single excitations for string I_β in active space $- 1$ **do**
        get t, u, sgn(tu), J_β such that $|\beta(J_\beta)\rangle = sgn(tu)\hat{E}^{\beta}_{tu}|\beta(I_\beta)\rangle$
        for $i \leftarrow 0,$ number of inactive orbitals $- 1$ **do**
          $F(J_\beta) \leftarrow F(J_\beta) + sgn(tu)(ii|tu)'$
      for $J_\beta \leftarrow 0,$ number of beta strings $- 1$ **do**
        for $I_\alpha \leftarrow 0,$ number of alpha strings $- 1$ **do**
          $\sigma_{3\_2}(I_\alpha, I_\beta, m) \leftarrow \sigma_{3\_2}(I_\alpha, I_\beta, m) + F(J_\beta)C(I_\alpha, J_\beta, m)$
